# Convolutional Neural Network-Based Technique for Gaze Estimation on Mobile Devices

**DOI:** 10.3389/frai.2021.796825

**Published:** 2022-01-26

**Authors:** Andronicus A. Akinyelu, Pieter Blignaut

**Affiliations:** Department of Computer Science and Informatics, Faculty of Natural and Agricultural Sciences, University of the Free State, Bloemfontein, South Africa

**Keywords:** Convolutional Neural Network, computer vision, gaze estimation, eye tracking, mobile device

## Abstract

Eye tracking is becoming a very popular, useful, and important technology. Many eye tracking technologies are currently expensive and only available to large corporations. Some of them necessitate explicit personal calibration, which makes them unsuitable for use in real-world or uncontrolled environments. Explicit personal calibration can also be cumbersome and degrades the user experience. To address these issues, this study proposes a Convolutional Neural Network (CNN) based calibration-free technique for improved gaze estimation in unconstrained environments. The proposed technique consists of two components, namely a face component and a 39-point facial landmark component. The face component is used to extract the gaze estimation features from the eyes, while the 39-point facial landmark component is used to encode the shape and location of the eyes (within the face) into the network. Adding this information can make the network learn free-head and eye movements. Another CNN model was designed in this study primarily for the sake of comparison. The CNN model accepts only the face images as input. Different experiments were performed, and the experimental result reveals that the proposed technique outperforms the second model. Fine-tuning was also performed using the VGG16 pre-trained model. Experimental results show that the fine-tuned results of the proposed technique perform better than the fine-tuned results of the second model. Overall, the results show that 39-point facial landmarks can be used to improve the performance of CNN-based gaze estimation models.

## Introduction

Eye tracking is a useful technology, and it can be applied to different domains, including medical diagnosis (Holzman et al., 1974), marketing (Wedel and Pieters, 2008), computer vision (Krafka et al., 2016), and human-computer interaction (Jacob and Karn, 2003). Many eye tracking systems exists, and some of them are expensive to purchase (Cazzato et al., 2020) or inaccurate (Wedel and Pieters, 2008). Some of them are also restrictive because they limit the functionalities available to users. These limitations have made eye tracking systems unavailable to many users. It has also made eye tracking research challenging to interested academics. In view of this, many researchers are designing and developing eye tracking systems that are affordable and available to users.

Eye tracking or gaze estimation studies have been primarily constrained to controlled environments (Kothari et al., 2020). Much work has not been done in the development of gaze estimation techniques for uncontrolled environments (Kothari et al., 2020). This study introduces a simple calibration-free Convolutional Neural Network (CNN) based technique for gaze estimation in mobile devices. The study aims to present a proof of concept for developing a CNN-based gaze estimation technique using full-face images and 39-point facial landmark images. CNN is used to extract gaze estimation features from full-face images and their corresponding 39-point facial landmarks as determined by the dlib library (McKenna and Gong, 1998). The 39-point facial landmark is used to encode the shape and location of the eyes into the network. We used the pre-trained facial landmark detector inside the dlib library (McKenna and Gong, 1998) to extract the facial landmarks. Specifically, we used the detector to estimate the location of 39 (*x, y*) coordinates that correspond to the shape of the face, left eye and right eye. The dlib facial landmark detector was originally designed to estimate 68 (*x, y*) coordinates that maps to different regions on the face, including left eye, right eye, nose, jaw, mouth, and the face region. It is an implementation of the method designed by Kazemi and Sullivan (2014). We did not use the entire 68 (*x, y*) coordinates because we are only interested in encoding the shape and location of the left and right eyes into the network, not the nose, jaw, or mouth. The proposed technique was evaluated, and it produced satisfactory results.

## Related Work

Different gaze estimation techniques have been proposed in the literature. Vora et al. (2017) developed a CNN-based technique for gaze detection. The technique consists of two units: the pre-processing unit and fine-tuning unit. The pre-processing unit was used to extract Region of Interest (ROI) images for training, including full-face images and upper-half face images. The extracted images were passed to the second unit for fine-tuning. The fine-tuning unit consists of two pre-trained models, namely: AlexNet (Krizhevsky et al., 2012), and VGG 16 (Simonyan and Zisserman, 2014). During experiments, the two pre-trained models were fine-tuned separately, and results show that the VGG-16 model produced an accuracy of 93.36%, when comparing predicted data with a test data set. The AlexNet model produced an accuracy of 88.91%. Naqvi et al. (2018) introduced a CNN-based technique for eye tracking in automobiles. The technique consists of one near-infrared camera, six near-infrared (NIR) light-emitting diodes (LEDs) for illumination, and one zoom lens. The NIR camera is used to capture the frontal view image of a driver. ROI images were extracted from the captured images, including face images, left eye images, and right eye images. The three ROI images were used to fine-tune three separate VGG-16 pre-trained models. The output from the three models were combined and used to estimate the gaze zone of a driver. The proposed technique was evaluated using two measures, namely: strictly correct estimation rate (SCER) and loosely correct estimation rate (LCER). Experimental results show that it produced an average SCER and LCER of 92.8 and 99.6%, respectively.

Krafka et al. (2016) proposed a CNN-based gaze estimation technique for eye tracking on mobile devices (called iTracker). They also introduced a large-scale dataset (called GazeCapture). The dataset contains over 2.5 million images from ~1,500 subjects. The dataset was used to build the CNN-based model with crops of the face, left eye, right eye and face grid (a binary mask that indicates the size and location of the head within the frame). Experimental results obtained from the study showed that the proposed model achieved a prediction error of 1.71 cm on mobile phones (~2.4° on a 6.4″ phone at 40 cm distance) and 2.53 cm on tablets (~2.9° on a 10.4″ tablet at 50 cm distance). The same technique was re-evaluated after simulating the process of calibration. Experimental results showed that it achieved a reduced prediction error of 1.34 cm on mobile phones and 2.12 cm on tablets. Kim et al. (2016) proposed a similar technique for mobile devices using CNN. They introduced a new feature called histogram-of-gradients (HOG) which was computed from cropped images of the face. The computed feature was combined with four other inputs: face image, left eye image, right eye image and face grid. The combined inputs were used to build a CNN model. The model was evaluated and it produced a prediction error of 4.85 cm (~7°) on iPhones.

Wang et al. (2016) proposed a calibration-free regression-based deep CNN that learns image features to predict eye fixations. In the study, a stochastic calibration approach was introduced. The approach aims to minimize the differences between the probability distribution of the predicted eye and the probability distribution of the actual eye gaze. It uses a deep fixation map obtained from Regression based CNN (RCNN) and a gaze distribution procedure to implicitly estimate personal eye parameter. The technique was evaluated and it was declared to produce satisfactory results. In a different study, Chen and Ji (2011) proposed a calibration-free technique using saliency maps. Saliency maps represent unique features of images. The technique was designed with the underlying assumption that users have a higher probability of looking at the salient regions of an image. The authors (Chen and Ji, 2011) designed a Bayesian network to represent the probabilistic relationship between the visual axis, optical axis and eye parameters. In the probabilistic model, a saliency map was used as the initial gaze input. A dynamic Bayesian network was also introduced to incrementally update the eye parameters online. Chen and Ji (2014) extended their study to handle Gaussian distribution. However, Gaussian distribution requires large-scale data before gaze point distribution can be approximated (Wang et al., 2016).

Bao et al. (2021) proposed a novel method for gaze estimation in mobile tablets called Adaptive Feature Fusion Network (AFF-Net). They layered channel-wise feature maps from the two eyes. Then, using Squeeze-and-Excitation layers, they adaptively fused the features of the two eyes depending on their appearance resemblance. The authors also proposed an adaptive group normalization method for recalibrating ocular features using facial cues as guidance. The approach was evaluated using the GazeCapture and MPIIFaceGaze datasets, with an error of 1.62 cm for GazeCapture and 3.9 cm for the MPIIFaceGaze dataset. Jigang et al. (2019) introduced the GazeEstimator, a two-step training network for enhanced gaze estimation on mobile devices. The first stage is to train a network for eye landmark localization on the 300W-LP dataset with the goal of properly localizing the eye on the image. The second phase involves training a gaze estimation network using the GazeCapture dataset to create a robust gaze estimation model. The approach was tested on the GazeCapture dataset and yielded a 1.25 cm in accuracy. Guo et al. (2019) introduced a new training scheme for CNN called tolerant and talented (TAT) training scheme. The training plan was created to address the issue of overfitting in CNN. It is an iterative approach for distilling random knowledge that incorporates cosine similarity pruning and aligned orthogonal initialization. An improved metric for evaluating the robustness of gaze estimators was also proposed by the authors. The proposed approach was tested on the GazeCapture dataset and yielded a 1.77 cm inaccuracy. Julien (Adler, 2019) developed a neural network based technique for estimating gaze on mobile devices. They trained a Siamese neural network to predict the linear distance between two gaze points on a screen. The technique was evaluated on the GazeCapture dataset, and it achieved a high Euclidean distance error of 1.33 cm. Table 1 shows the summary of the related works considered in this study.

**Table 1:** Summary of related works.

**References**	**Model**	**Dataset**	**Performance**
Vora et al. (2017)	VGG16 and AlexNet	Own dataset	93.36% accuracy (VGG16) and 88.91% (AlexNet)
Naqvi et al. (2018)	VGG16	Own dataset	92.8% (SCER) and 99.6% (LCER).
Krafka et al. (2016)	Own architecture; AlexNet	GazeCapture	1.71 cm
Kim et al. (2016)	Gazelle architecture	GazeCapture	4.85 cm
Wang et al. (2016)	Own architecture (RCNN)	MIT1003 dataset	1.0 degree
Bao et al. (2021)	AFF-Net	GazeCapture and MPIIFaceGaze	1.62 cm (GazeCapture) and 3.9 cm (MPIIFaceGaze)
Jigang et al. (2019)	ResNet model	GazeCapture	1.25 cm
Guo et al. (2019)	Own model	GazeCapture	1.77 cm
Adler (2019)	Siamese neural network	GazeCapture	1.3 cm

## Proposed Technique

Currently, many eye tracking technologies are available, however the most of them are expensive and commercial (Krafka et al., 2016; Cazzato et al., 2020). Others have been designed in controlled environments, rendering them less reliable in real-world settings. Furthermore, most eye tracking technologies necessitate an explicit personal calibration approach to determine subject-dependent eye characteristics. Such calibration procedure is unnatural, inconvenient, and impairs user experience (Wang et al., 2016). To address these issues, this study presents a regression-based, calibration-free gaze estimation CNN model for improved eye tracking in an unconstrained environment.

**Figure 1:**
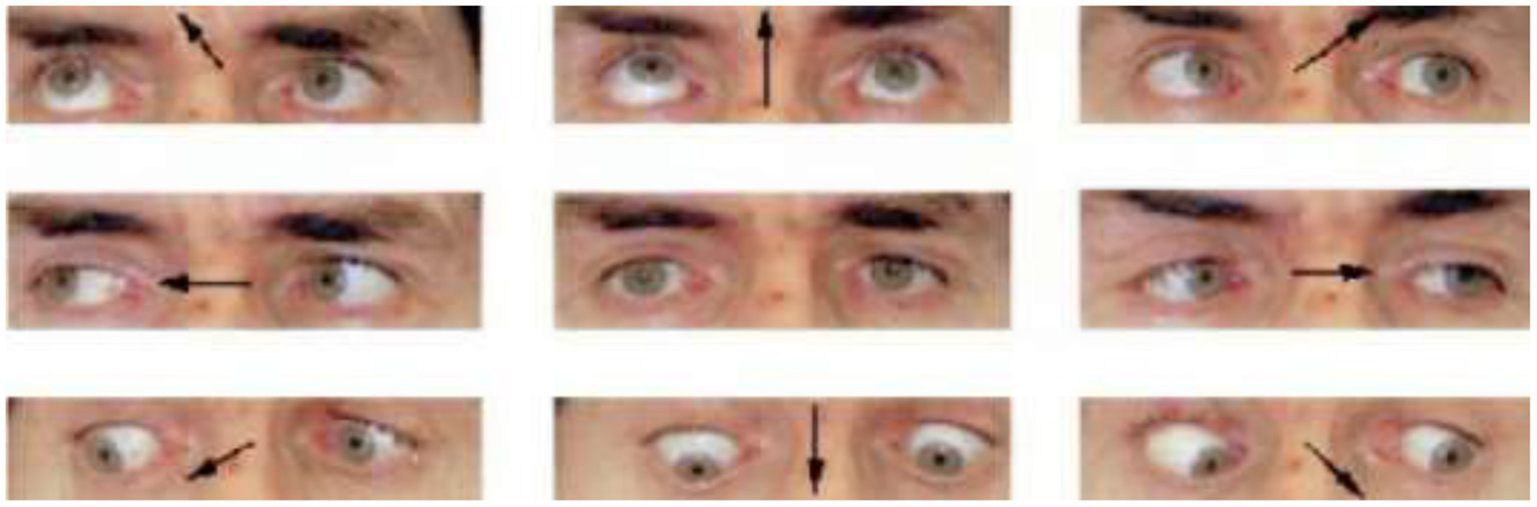
Different appearance of the eyes (Bejjani et al., 2002).

The proposed technique uses the face images and their corresponding 39-point facial landmarks [as acquired from the dlib library (McKenna and Gong, 1998)] to perform gaze prediction. The 39-point facial landmark is added to the CNN model with the goal of encoding the shape of the eyes and the location of the eyes (within the face) into the model. The shape and location of the eyes can provide information on where a person is looking at. As an example, if a user is looking at different points on a screen, the user's eyes will move around the screen. The movement is particularly obvious when the user is looking at different points randomly displayed at extreme corners of a screen (e.g., top-left, top-right, bottom-left, and bottom-right corners). The user may have to move his eyes (and sometimes his head) to look at the different points. The movements may change the shape of his/her eyes and the position of his/her head. Figure 1 shows the image of a user looking at different directions. As shown, the shape of the eyes changes when the user spontaneously moves his eyes in the directions specified by the arrow. The goal of the proposed technique is to capture these changes and encode them into a CNN model. Encoding these changes can improve the accuracy of gaze estimation systems.

The proposed technique consists of two components, namely, (i) a face component and (ii) a 39-point facial landmark component. A component refers to a set of inputs. The first component is used to extract gaze estimation features from the eyes, while the second component is used to extract features from the 39-point facial landmark. The 39-point facial landmark features can help the network learn free-head and eye movements. More details on the model's architecture are provided in section Model Architecture. Another CNN model (called Network-2) is designed in this study primarily for the sake of comparison. This model accepts only the face images as input. The main difference between the proposed technique and Network-2 is their network configuration. Network-2 does not have the 39-point facial landmark component. Meanwhile, the face component of the proposed technique and Network-2 has similar configuration. Their configuration is similar because we want to evaluate the impact of the 39-point facial landmark. We also want to ensure a fair comparison between the two models. A third CNN model was designed in this study (called Network-3). This model accepts only the 39-point facial landmarks as inputs. The model was designed with the goal of evaluating the performance of the 39-point facial landmark and its contribution to gaze estimation models.

CNN is good at transfer learning (Vora et al., 2017). Image representations learned from a large-scale labeled dataset can be efficiently transferred to other similar visual recognition tasks with limited amount of training datasets (Oquab et al., 2014). In this study, we fine-tuned the proposed technique using the VGG16 pre-trained network (Simonyan and Zisserman, 2014). The VGG16 model was originally trained on the ImageNet dataset containing over 14 million images belonging to 1,000 classes. During fine-tuning, we removed the last fully connected layer of the VGG16 network (which has 1,000 classes) and added some convolutional and fully connected layers to the VGG16 network. We also freeze the pre-trained weights of the VGG16 model. Finally, we trained the added layers using the training dataset. During training, the *x* and *y* ground truth labels were provided to the network.

## Methods

A deep learning task for gaze estimation can be considered as a regression or classification task. Although, both regression and classification tasks are useful, regression offers the highest predictive flexibility (Lemley et al., 2018). This study deals with gaze estimation as a regression task. The goal of the regression task is to find a gaze point (*x, y*) on a screen, which corresponds to where a user is looking at.

The CNN models designed in this study were implemented using Keras—an open-source neural network library written in Python programming language. The entire training process is divided into two stages, namely: (i) hyper-parameter search stage and (ii) evaluation stage. During the hyper-parameter search stage, different models are evaluated, and the model with the best hyper-parameter configuration is selected. This model is passed to the evaluation stage for training, validation, and testing.

At the hyper-parameter search stage, a Keras Tuner function was used to search for the best hyper-parameter configuration. The Keras Tuner function has two types of tuners, namely, Hyperband and RandomSearch tuners. In this study, the RandomSearch tuner was used. The tuner requires a model-building function where the network architectures and different ranges of hyper-parameter values are specified by a user. The tuner is instantiated and the search for the best hyper-parameter configuration is initiated. During the search, different models are built iteratively by calling the model-building function. The function populates the search space using the range of hyper-parameter values specified by the user. The tuner progressively explores the search space and records the performance for each network configuration. After the search, the model that produces the best result can be retrieved and fine-tuned for *n* epochs, where *n* is user-defined. In this study, hyper-parameter tuning was performed for all the networks. During the hyper-parameter search, all the evaluated models were trained for *n* epochs, where *n* = 3 for this study. After the search, we retrieved the best model (which is already trained for three epochs) and trained it for another seven epochs. Therefore, the total number of epochs used to train the entire model is 10 epochs.

### Dataset

The proposed technique was evaluated on two datasets, namely GazeCapture (Krafka et al., 2016) and TabletGaze (Huang et al., 2017). The GazeCapture dataset contains over 2 million images from 1,474 subjects. The subjects were required to do a dot-tracing task and their images along with the coordinates of the dot were captured using the front-facing camera of their devices. The following information were included in the GazeCapture dataset:

(a) The front-facing full-face images of the subjects as they perform the dot tracing task.
(b) The bounding box coordinates for the face and eye images in the full-face image.
(c) The ground truth gaze coordinates (that is, the actual gaze coordinates in the *x* and *y* direction). The *x* coordinate indicates the distance (in cm) to the left or right direction of a camera on a virtual plane that contains the true location, while the *y* coordinate specifies the distance in the up and down direction of a camera. This coordinate system permits a model to estimate gaze coordinates that can be generalized to multiple devices (such as laptops and smartphones) and orientations (portrait or landscape), depending on where the camera is positioned on the screen. It takes advantage of the fact that the front-facing camera is typically on the same plane to the screen, and it is angled perpendicular to the screen (Krafka et al., 2016; Akinyelu and Blignaut, 2020).

Based on the three information points outlined above, we extracted the face images from the frames in the datasets. The TabletGaze dataset contains 816 videos from 51 subjects. During the data collecting session performed by the dataset providers (Huang et al., 2017), each subject held a tablet in one of four body postures: standing, sitting, slouching, or lying. Each participant was asked to undertake four recording sessions for each of the four body postures, resulting in a total of sixteen video sequences for each subject. In addition, each subject was required to look at 35 different gaze points during the recordings. As indicated by Huang et al. (2017), not all the videos in the dataset are usable, therefore, in this study, we focused on videos where the whole face is visible. To eliminate the time required for participants to refocus on each dot location, similar to Huang et al. (2017), we extract only the video chunk that corresponds to 1.5 to 2.5 s after the dot appears in a new location.

**Figure 2:**
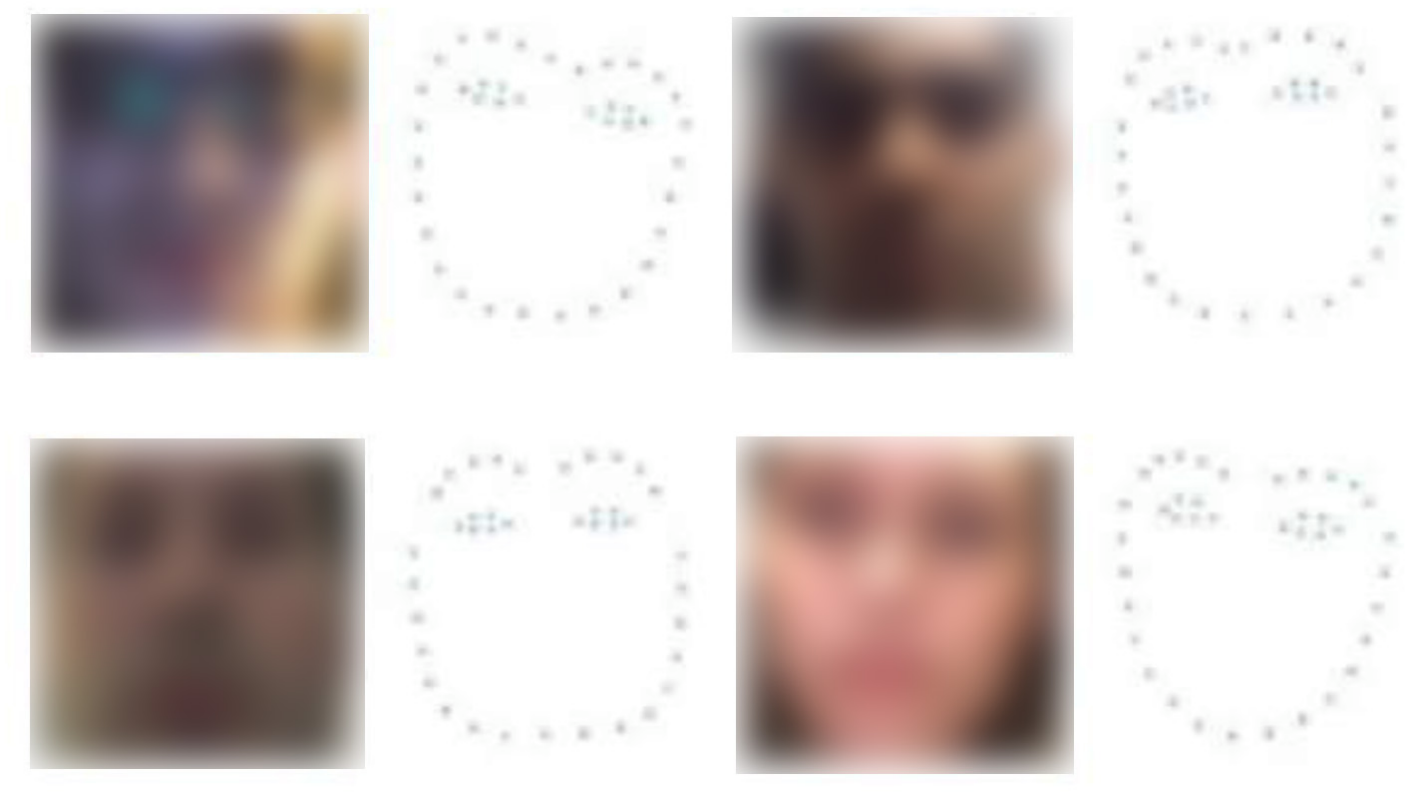
Sample images of face and 39-point facial landmark.

We also extracted the 39-point facial landmarks from the frames in the two datasets using the pre-trained facial landmark detector inside the dlib library (McKenna and Gong, 1998). Some samples of the face images and their corresponding 39-point facial landmark images are shown in Figure 2.

This paper aims to present a proof of concept for developing a CNN-based gaze estimation technique using full-face images and 39-point facial landmark images. In view of this, we used only a subset of the GazeCapture dataset for our experiments. The subset consists of 31,920 images from 11 subjects (15,960 full face images and 15,960 39-point facial landmark images). We also used a subset of the TabletGaze dataset. The subset consists of 27,848 images from 20 subjects (13,924 full face images and 13,924 39-point facial landmark images).

### Input to the Convolutional Neural Network

The images in the GazeCapture dataset were of dimension 480 × 640. Similar to Kim et al. (2016), we wrote some scripts to crop the full-face images from the original frames and resized them to 224 × 224. We also wrote some scripts to extract the 39-point facial landmarks from the cropped face images and resized them to 224 × 224. All the images were normalized to the range [0, 1]. The labels in the datasets (i.e. *x* and *y* gaze coordinates) were also normalized to the range [0, 1]. Finally, the images and their corresponding labels were used to build the models. Eighty percent of the dataset was used for training, while the remaining 20% was used for validation and testing.

### Model Architecture

**Figure 3:**
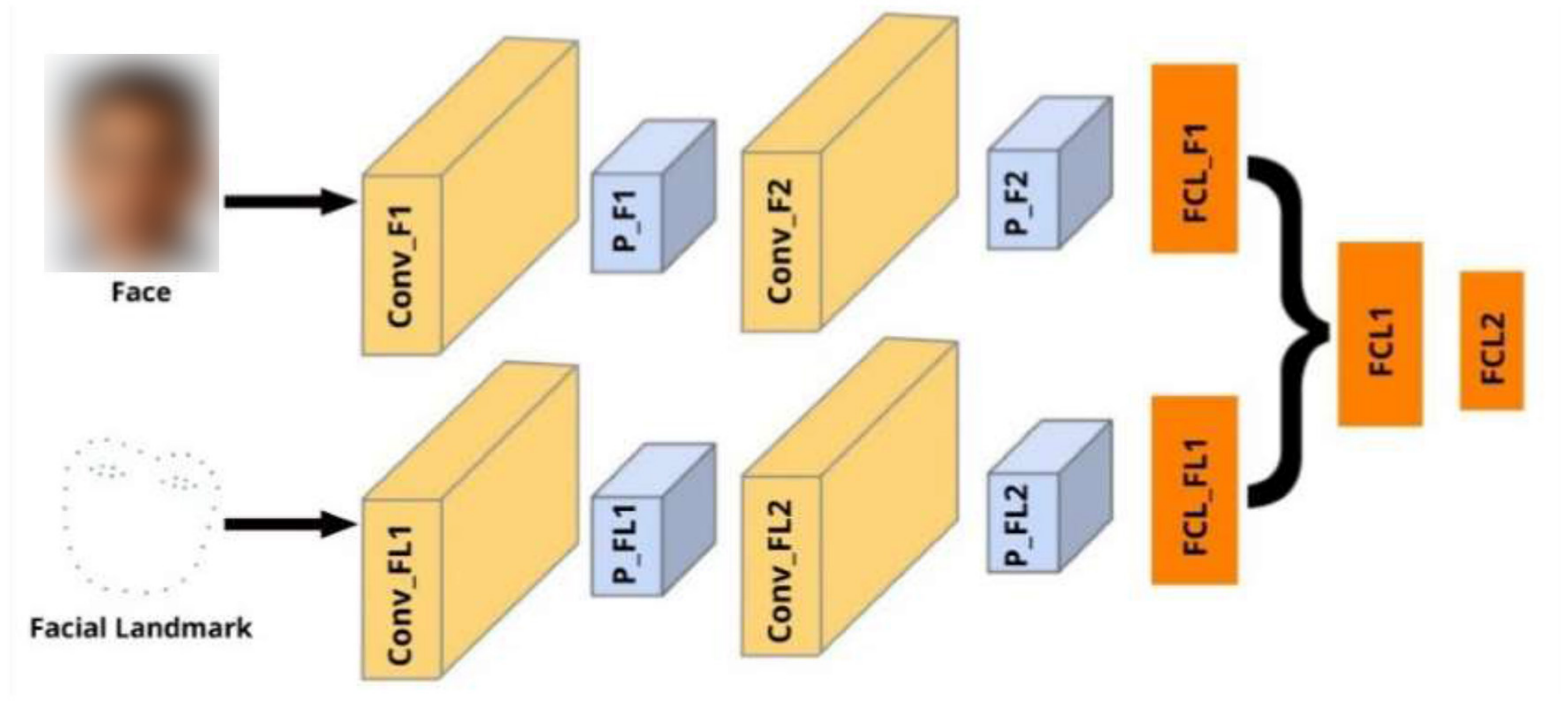
Overview of the proposed technique. Inputs include face and facial landmark, all of size 224 × 224. Conv refers to convolutional layers, P represents pooling layer, FCL represents Fully Connected layers. FCL2 is the output layer.

The network architecture for the proposed technique is divided into two components. The first component is used to process the full-face images, while the second component is used to process the 39-point facial landmarks. As shown in Figure 3, the first component consists of two convolutional layers and one fully connected layer, while the second component consists of two convolutional layers and one fully connected layer. In both components, each convolutional layer is followed by an average pooling layer. The fully connected layer of the first and second components are concatenated and passed through another fully connected layer and one output layer. The output layer contains two neurons, which represents the estimated *x* and *y* gaze coordinates. We used the rectified linear activation function (ReLU) for all the layers, except the output layer. We used the linear activation function for the output layer because we are working on a regression problem (i.e., gaze estimation). The overview of the proposed technique is shown in Figure 3.

As noted in section Proposed Technique, we designed another CNN model (i.e., Network-2) for the sake of comparison. Network-2 accepts face images as input. It consists of two convolutional layers, one fully connected layer and one output layer. Each convolutional layer is followed by an average pooling layer. The output layer contains two neurons. The ReLu activation function is used for all the layers except the output layer, where we used the linear activation function.

We performed fine-tuning for the proposed technique using the VGG16 pre-trained model. The fine-tuning model consist of two components. The first component is used to process the face images using the VGG16 pre-trained model. The VGG16 network accepts the face images as input. The output layer of the VGG16 network is removed and passed through one average pooling layer. The average pooling layer is followed by one convolutional layer, one average pooling layer, one fully connected layer, and one dropout layer. The dropout layer is used to prevent overfitting. The dropout rate is set to 0.5. The second component is used to process the 39-point facial landmark images. It consists of one convolutional layer, followed by one average pooling layer, and one fully connected layer. The output from the dropout layer of the first component is concatenated with the output from the fully connected layer of the second component. The concatenated output is passed through one fully connected layer and one output layer, containing two neurons. Similar to other experiments, we used the ReLU activation function for all the layers, except the output layer where we used the linear activation function. During fine-tuning, we freeze all the pre-trained layers (excluding the output layer), and trained only the added layers.

We also performed fine-tuning for Network-2 using the VGG16 pre-trained model. During fine-tuning, we removed the output layer of the VGG16 network and passed it through one average pooling layer. The average pooling layer is followed by one convolutional layer, one average pooling layer, one fully connected layer, one dropout layer, and one output layer. The dropout rate is set to 0.5.

### Evaluation Metrics

The proposed technique was built and trained from the scratch. The hyper-parameters used for training are reported in Table 2. In the Table, beta_1 and beta_2 refers to the exponential decay rate for the 1st and 2nd moment estimates respectively. Similar to No Matches Found, we report the error in terms of the Average Euclidean Distance (AED) from the point of true fixation [see equation (1)]. The AED is reported in centimeters and degrees. We also report the accuracy and Mean Square Error (MSE) produced by the models.


(1) $$\begin{array}{l} \text{Average Euclidean distance}\\ =\frac{1}{n}\sum_{i=1}^{n}\sqrt{{\left({gt\text{\_}x}_{i}-\;{e\text{\_}x}_{i}\right)}^{2}+\;{\left({gt\text{\_}y}_{i}-\;{e\text{\_}y}_{i}\right)}^{2}} \end{array}$$

where *gt*_*xi and gt*_*yi* refers to the ground truth label for each input, and *e*_*xi and e*_*yi* refers to the estimated (*x, y*) gaze coordinates for each input.

**Table 2:** Hyper-parameter values for training.

**Hyper-parameters**	**Value**
Epochs	10
Learning rate	0.001
Batch size	16
Optimizer	Adam
beta_1	0.9
beta_2	0.999
Loss function	MSE

## Result and Discussion

Different experiments were performed to evaluate the performance of the proposed technique. Table 3 shows the AED, MSE, and accuracy produced by the proposed technique. The table also shows the performance of the second model (i.e., Network-2) designed in this study for the sake of comparison. The proposed technique was trained on face images and their corresponding 39-point facial landmark images, while Network-2 was trained on face images only. As shown in the result, the proposed technique outperformed Network-2, achieving an AED of 0.22 cm and a MSE of 0.0378 (~0.32° on a 4.7″ phone at 40 cm distance). This shows that the 39-point facial landmarks improved the performance of the model.

**Table 3:** Results produced by the proposed technique for GazeCapture dataset.

**Model**	**Average Euclidean distance (cm)**	**MSE**	**Accuracy (%)**
Face + FL (Proposed technique)	0.2221 (~0.32° at 40 cm distance)	0.0378	94.2042
Face only (Network-2)	0.2468 (~0.35° at 40 cm distance)	0.0436	93.4210
Face + FL + VGG16 (Proposed technique)	0.1278 (~0.19° at 40 cm distance)	0.0116	96.8358
Face + VGG16 (Network-2)	0.1358 (~0.20° at 40 cm distance)	0.0127	95.3947
FL only (Network-3)	0.3228 (~0.46° at 40 cm distance)	0.0761	89.0664
FL + VGG16 (Network-3)	0.3119 (~0.45° at 40 cm distance)	0.0733	89.6303

*Key FL, 39-point Facial Landmark; MSE, Mean Square Error*.

More experiments were performed to evaluate the performance of the 39-point facial landmarks. In the experiments, the face component of the proposed technique was removed, and the facial landmark component was trained on the 39-point facial landmark images. The results obtained are reported in Table 3. The model produced an AED of 0.32 cm (~0.47°) and a MSE of 0.0761. As observed, the full model performed better than the facial landmark model, but the performance of the facial landmark model is still acceptable. This shows that the 39-point facial landmarks has the potential to be used as a standalone input for developing improved gaze estimation models. It also shows that the 39-point facial landmarks can be combined with other inputs for improved gaze estimation.

More experiments were performed to improve the performance of the proposed technique using transfer learning. Specifically, the VGG16 pre-trained model was used to fine-tune the performance of the proposed technique. We performed fine-tuning for both the proposed technique and the compared technique (i.e., Network-2). The fine-tuned results are reported in Table 2. As shown in the Table, the fine-tuned result of the proposed technique outperformed the fine-tuned result of Network-2. It achieved an AED of 0.13 cm and MSE of 0.0116 (~0.19° on a 4.7″ phone at 40 cm distance). This further shows the significance of the 39-point facial landmarks. The result also shows that the fine-tuned results of the proposed technique outperform the results of the main model. This shows the importance of fine-tuning in reducing error.

**Figure 4:**
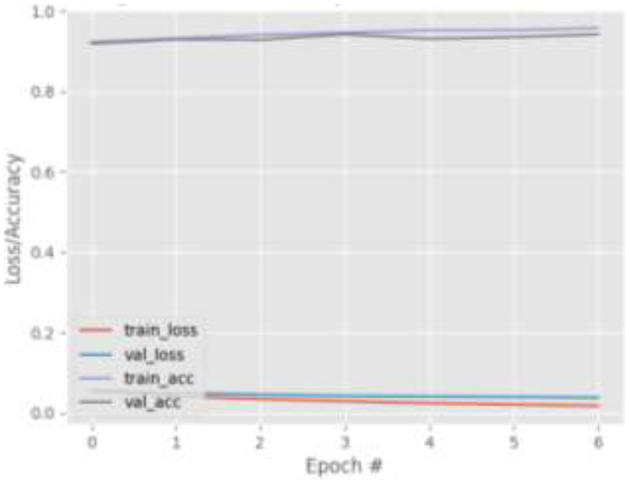
Results for Face + FL on GazeCapture dataset.

**Figure 5:**
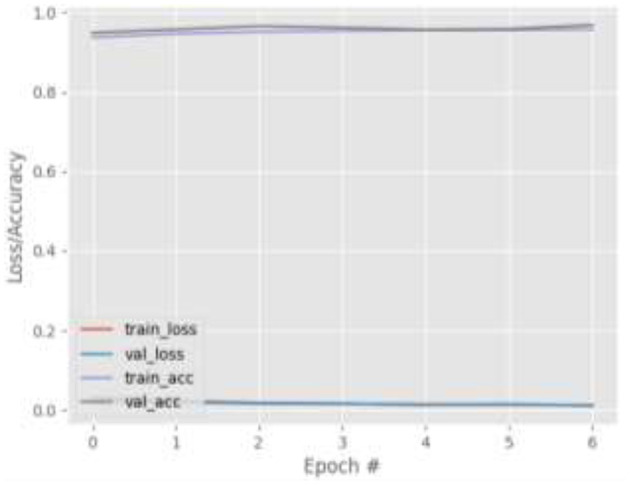
Results for Face + FL + VGG16 on GazeCapture dataset.

**Figure 6:**
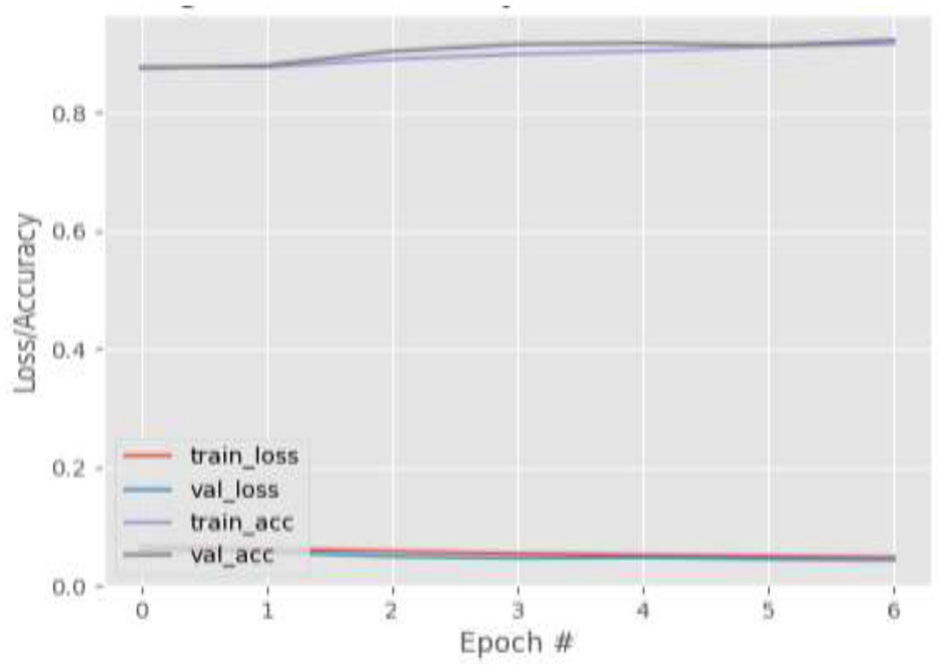
Results for FL only (39-points facial landmarks) on GazeCapture dataset.

**Figure 7:**
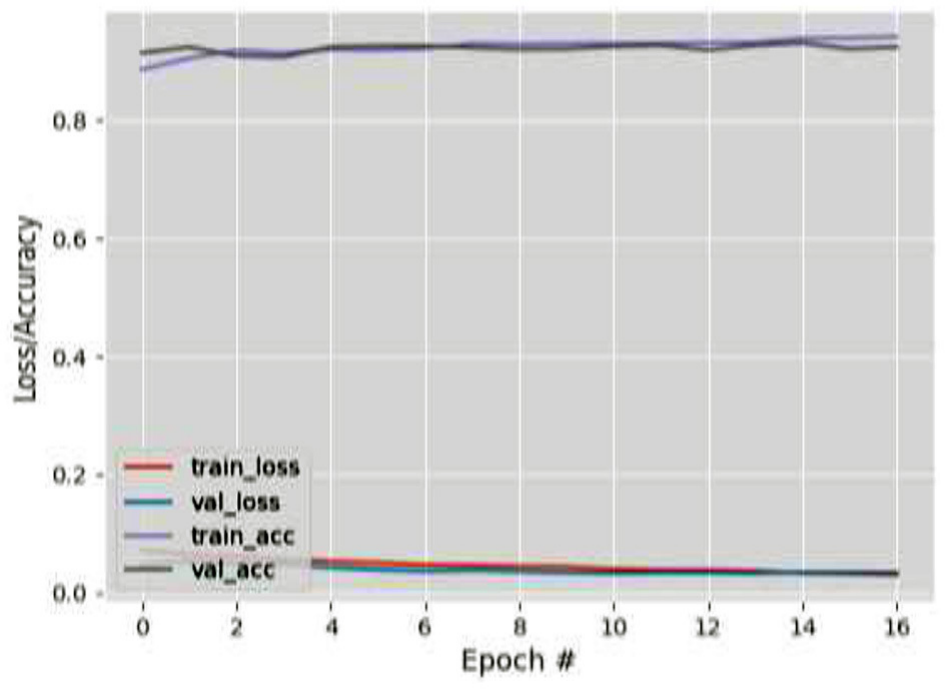
Results for FL + VGG16 on GazeCapture dataset.

Figures 4–7 show the training and validation loss (i.e., MSE) produced by the proposed technique for the GazeCapture dataset. The figures also show the training and validation accuracies produced by the proposed technique. As explained in section Methods, the entire training process is divided into two stages. At the hyper-parameter search stage, the models were trained for three epochs. At the end of this stage, the best model was selected and trained (at the second stage) for another seven epochs. Figures 4–7 show the performance of the models at the second stage, which is the main training stage. As shown in the figures, there is no large discrepancy between the training and validation loss produced in each epoch. There is also no large discrepancy between the training and validation accuracy produced in each epoch. This shows that there is no overfitting in the trained models. It also shows the generalization performance of the proposed technique.

**Figure 8:**
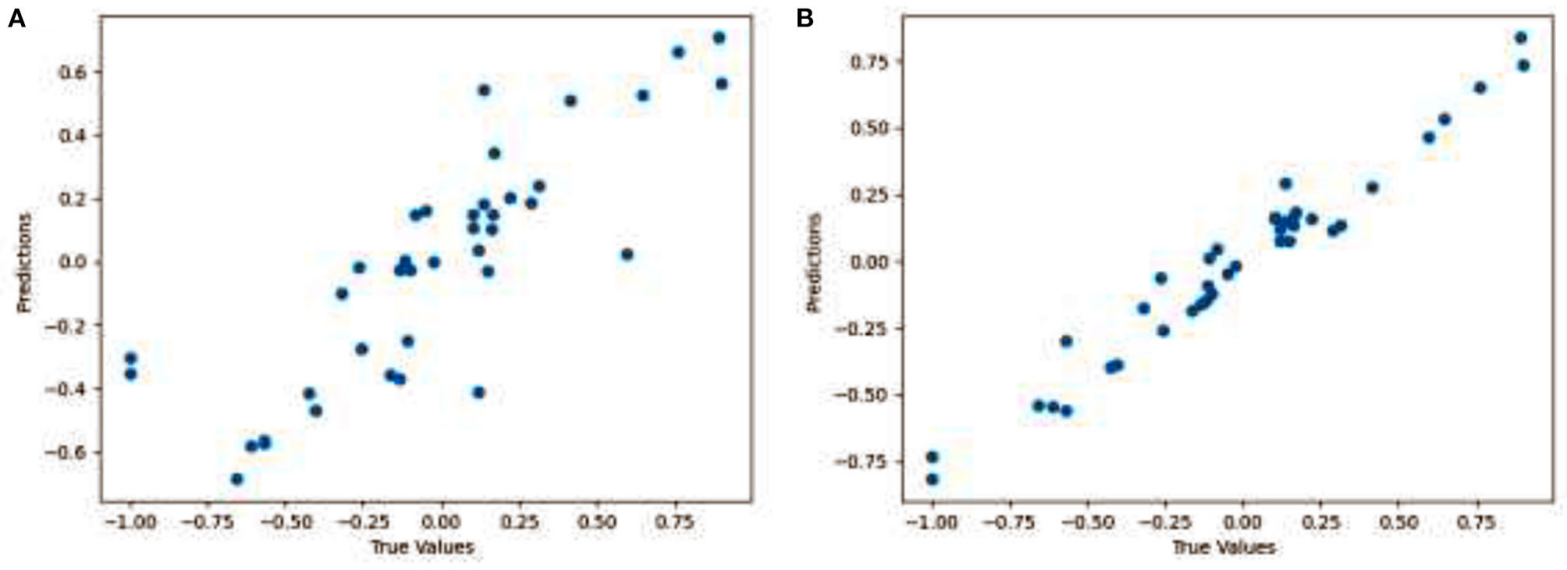
Visualization showing the distance between the ground truth and the predicted gaze for **(A)** Face + FL and **(B)** Face + FL + VGG16.

Figure 8 shows the distance between the ground truth and the predicted gaze values. Figure 8A shows the results for the proposed technique (Face + FL), while the Figure 8B shows the results for the finetuned proposed technique (Face + FL + VGG16). We reported findings for only 20 pairs of gaze locations to avoid overcrowding the plot and to guarantee that it is easily comprehendible. As can be seen from the two plots, the gaze estimations for each gaze position in the figures are very near to the ground truth gaze locations. Figure 8B also demonstrates that fine-tuning the proposed technique contributed to a reduction in gaze estimation error. The results illustrate that, on average, the proposed technique yields a satisfactory Euclidean distance.

As indicated previously, the proposed approach was evaluated on the TabletGaze dataset. Table 4 summarizes the AED, MSE, and accuracy of the proposed technique (face + FL) and compared techniques (face only). As can be observed, the proposed technique slightly outperformed the compared technique in terms of AED and MSE. The fine-tuned version of the proposed technique (Face + FL + VGG16) also outperformed the compared technique by a little margin. This illustrates the role of the 39-point face landmark in improving the model's performance. Another experiment was performed to evaluate the performance of the 39-point facial landmark. The 39-point facial landmark was utilized to train another CNN model, and the results were better than when the model was trained on the face only, as shown in Table 4.

**Table 4:** Results produced by the proposed technique for TabletGaze dataset.

**Model**	**Average Euclidean distance (cm)**	**MSE**	**Accuracy (%)**
Face + FL (Proposed technique)	0.3510	0.07145	70.95153
Face only (Network-2)	0.35365	0.07255	70.95153
Face + FL + VGG16 (Proposed technique)	0.35275	0.07199	70.95153
Face + VGG16 (Network-2)	0.36102	0.07454	72.70454
FL only (Network-3)	0.34971	0.07091	70.95153
FL + VGG16 (Network-3)	0.3500	0.0710	70.9515

*Key FL, 39-point Facial Landmark; MSE, Mean Square Error*.

Krafka et al. (2016) also examined their technique on the TabletGaze dataset, and the study's findings indicate that the dataset did not perform as well as the GazeCapture dataset. Similarly, in this study, the proposed technique did not yield satisfactory results for the TabletGaze dataset when compared to the GazeCapture dataset. It attained an accuracy of 94.2042% when evaluated on the GazeCapture dataset, but only 70.9515% when evaluated on the TabletGaze dataset. The reason for this warrants more investigation. Nevertheless, the GazeCapture dataset appears to be a better dataset for gaze estimation, based on the results.

**Figure 9:**
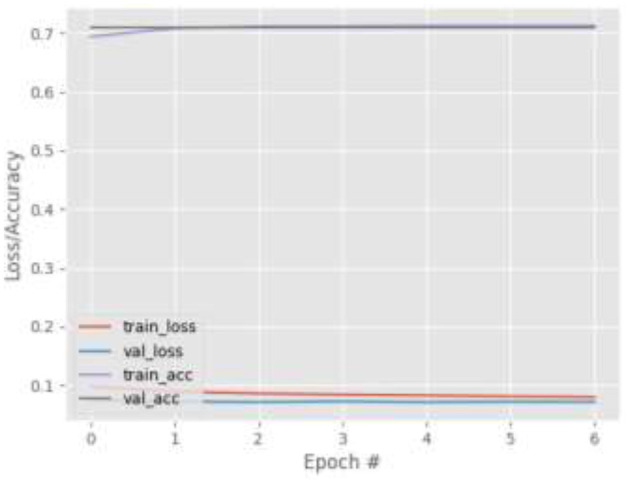
Results for Face + FL on TabletGaze dataset.

**Figure 10:**
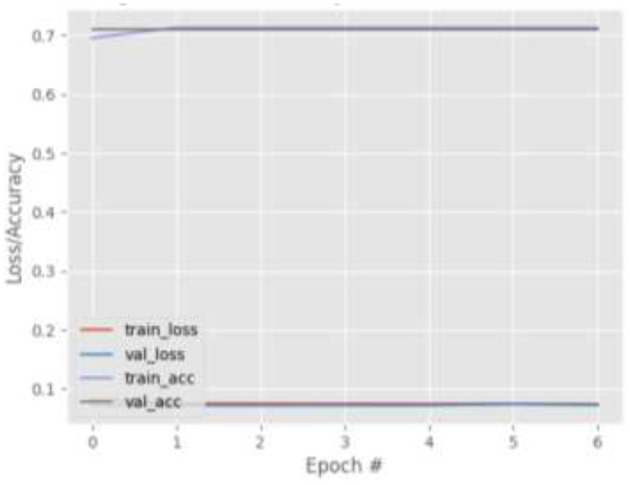
Results for Face + FL + VGG16 on TabletGaze dataset.

**Figure 11:**
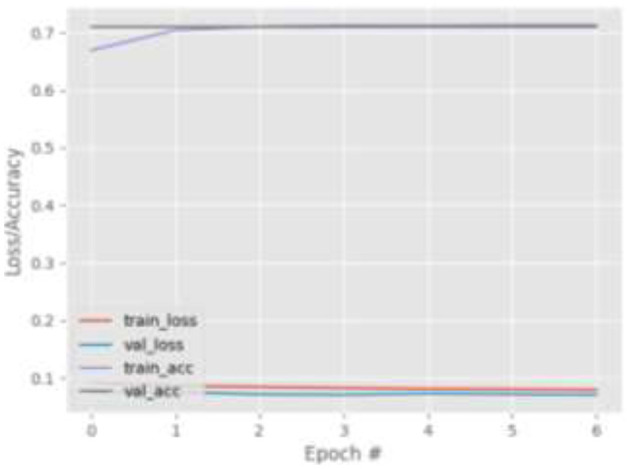
Results for FL only (39-points facial landmarks) on TabletGaze dataset.

**Figure 12:**
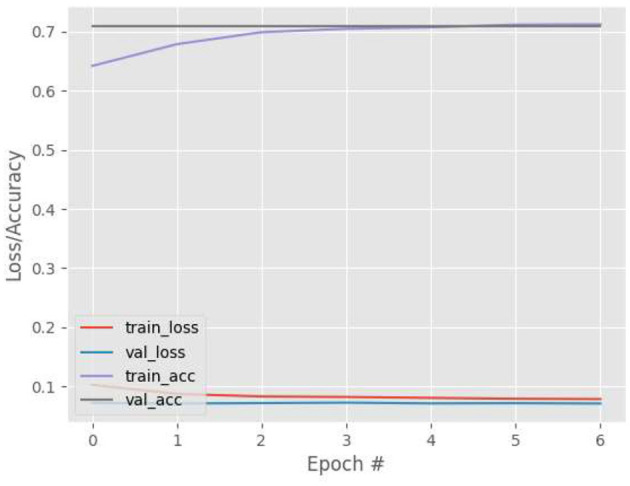
Results for FL+ on TabletGaze dataset.

Figures 9–12 show the training and validation loss (i.e., MSE) produced by the proposed technique for the TabletGaze dataset. The figures also show the training and validation accuracies produced by the proposed technique. As can be observed, while the accuracy and AED of the models is not very high, their generalization performance is adequate. There is no significant difference in the training and validation losses generated in each period. Additionally, there is no significant difference between the accuracy achieved by training and validation in any period. This shows that there is no overfitting in the model.

**Figure 13:**
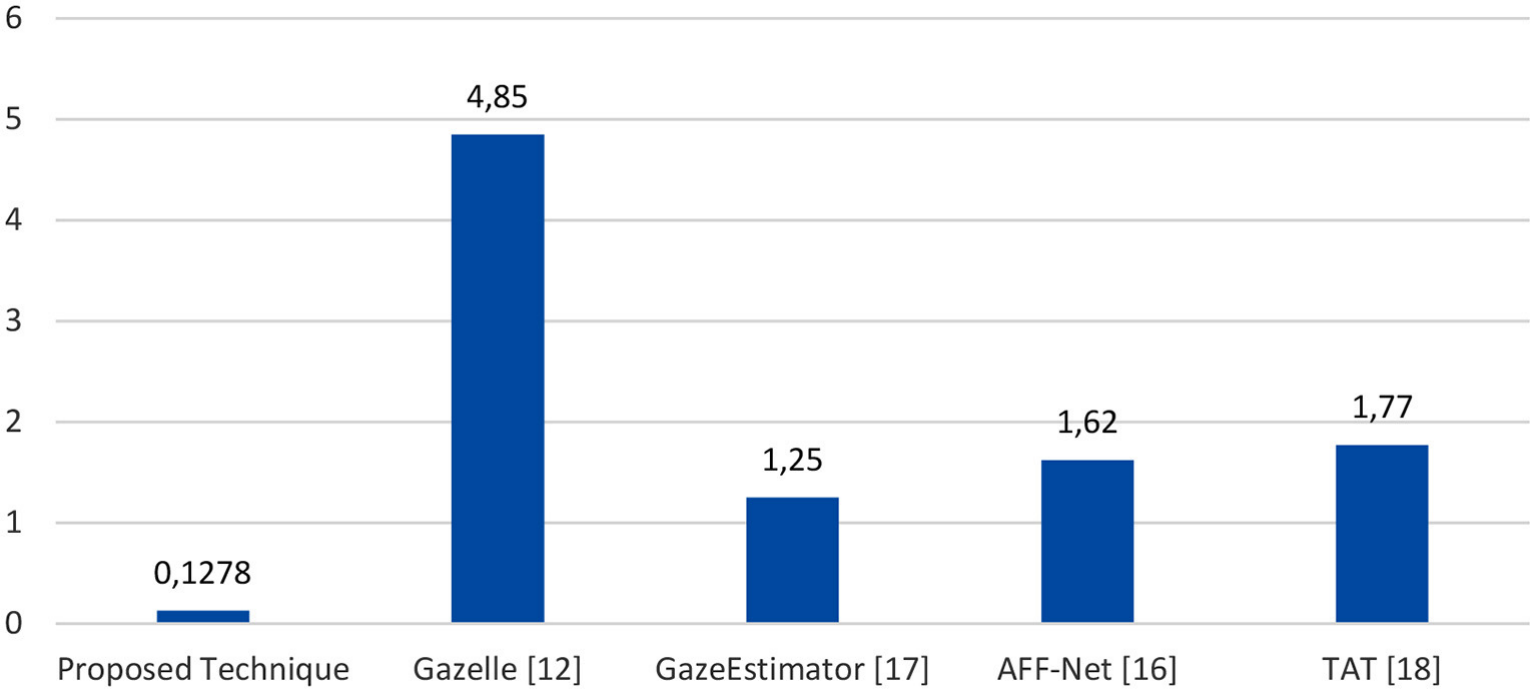
Comparison between proposed technique and four techniques.

The proposed technique (Face + FL + VGG16) is compared against four previously published algorithms on the GazeCapture dataset: GazeEstimator (Jigang et al., 2019), Gazelle (Kim et al., 2016), AFF-Net (Bao et al., 2021), and TAT (Guo et al., 2019). Table 5 and Figure 13 shows the outcomes of the four techniques. As shown, the proposed technique outperforms the four compared techniques. It achieved the best Euclidean distance of 0.1278 cm, followed by GazeEstimator and AFF-Net.

**Table 5:** Proposed technique vs other techniques.

**Model**	**Euclidean distance (cm)**
Proposed technique (Face + FL + VGG16)	0.1278
Gazelle (Kim et al., 2016)	4.85
GazeEstimator (Jigang et al., 2019)	1.25
AFF-Net (Bao et al., 2021)	1.62
TAT (Guo et al., 2019)	1.77

*Key FL, 39-point Facial Landmark*.

## Summary

This paper introduces a CNN-based calibration-free technique for improved gaze estimation. The technique accepts two inputs, namely: full-face images and 39-point facial landmark images. The 39-point facial landmark is used to encode the shape and location of the eyes and head into the network. Different experiments were performed, and the results show that the proposed technique produced good results. It also shows that the 39-point facial landmarks improved the performance of the proposed gaze estimation model. The performance can be further improved by training the proposed technique on a larger dataset. This is our plan for future research.

## Data Availability Statement

Publicly available datasets were analyzed in this study. This GazeCapture dataset can be found here: https://gazecapture.csail.mit.edu/download.php, https://github.com/CSAILVision/GazeCapture. The TabletGaze dataset can be found here: https://rice.app.box.com/s/nvc48slwmrtrmnmnpmg1l0pyp5f7pbow.

## Author Contributions

All authors listed have made a substantial, direct, and intellectual contribution to the work and approved it for publication.

## Funding

The authors would like to express their gratitude to the University of the Free State for paying the article processing fees associated with this work.

## Conflict of Interest

The authors declare that the research was conducted in the absence of any commercial or financial relationships that could be construed as a potential conflict of interest.

## Publisher's Note

All claims expressed in this article are solely those of the authors and do not necessarily represent those of their affiliated organizations, or those of the publisher, the editors and the reviewers. Any product that may be evaluated in this article, or claim that may be made by its manufacturer, is not guaranteed or endorsed by the publisher.
